# Gas accumulation in Lake Bosumtwi deep waters and its potential to contribute to fish kills

**DOI:** 10.1007/s11356-025-36032-z

**Published:** 2025-02-10

**Authors:** Bertram Boehrer, Tom Shatwell, Asha Damoah, Patrick Aurich, Maria Determann, Peter Sanful, Wolf von Tümpling

**Affiliations:** 1 Helmholtz Centre for Environmental Research—UFZ, Magdeburg, Germany; 2 University of Energy and Natural Resources (UENR), Sunyani, Ghana

**Keywords:** Gas accumulation, Tropical lake, Fish kills, Meromixis, Stratification, Bound nitrogen

## Abstract

Lake Bosumtwi in tropical Ghana has been known for its recurrent fish kills, but they have recently been reported to happen less frequently. The lake formed in a meteorite impact crater in Ghana, West Africa. It plays an important role for the local inhabitants for recreation and for fisheries. The lake is deep, and recent observations indicate that recirculation is incomplete. In general, the deep water is anoxic. Fish kills have been associated with the mixing events in the slightly colder rainy season. As unpleasant smells from the water during deep mixing had been reported, the question arose whether toxic gases that had accumulated in the deep water could be responsible; namely, hydrogen sulphide or large amounts of carbon dioxide were considered the most probable candidates. The analysis of the water properties, however, did not detect any hydrogen sulphide nor immensely large concentrations of carbon dioxide. On the contrary, the presence of large amounts of bound nitrogen could be substantiated. We hence concluded that most probably bound nitrogen was responsible for the fish kills on two paths (1) as bound nitrogen as ammonium forms toxic ammonia when mixed into high pH surface water and (2) depletes oxygen when it is oxidized in the surface waters.

## Introduction

The water quality in lakes is heavily impacted by the stratification and circulation pattern (e.g. Imboden and Wüest 1995; Lerman et al. 1995; Boehrer and Schultze 2008). During stratification periods, epilimnion and deep water develop differently: while the surface water is in continuous exchange with the atmosphere, the deep water is excluded from further exchange with the atmosphere (e.g. Schwoerbel 1999; Wetzel 2001; von Rohden et al. 2010; Boehrer et al. 2021a). Substances can accumulate in the deep water until the next deep recirculation (e.g. Findenegg 1935; Schmid et al. 2004; Boehrer et al. 2014, 2017; Gulati et al. 2017). Those substances can be unpleasant or even represent a danger when deep recirculation transports them into the surface water.

Some lakes do not circulate to the deepest spot and, as a consequence, develop a chemically different deep water layer. These lakes are called meromictic in contrast to holomictic lakes, which circulate completely usually at least once a year. The understanding of mixing types is strongly tied to studies in temperate climates. Though there have been studies on lake in the tropical and sub-tropical zone already in the 1980s (e.g. Melack 1984), their circulation behaviour has until now been much less studied and the numerical models are not yet designed for covering the special features of tropic lakes yet. In temperate climates, lake surfaces are exposed to a large temperature amplitude over the seasons, which is responsible for forming and breaking down the density stratification and the resulting clear circulation and stratification patterns. Usually, circulation depth can be clearly identified from oxygen profiles or steps in the profiles of electrical conductivity (e.g. Boehrer et al. 2014), but there are also more difficult cases.

The situation can be quite different in tropical lakes. Though some lakes are also clearly identifiable meromictic lakes in the tropics like Lake Kivu with typical features that are unique to meromictic lakes (e.g. Newman 1976; Kling et al. 1987; Schmid et al. 2005, 2019), there are also tropical lakes that may not overturn completely and have deep regions where oxygen is never detectable, yet deep water is still renewed by episodic events such as surface cooling and plume formation. In addition, even if the deep recirculation has not reached the deepest point of the lake, vertical exchange in the weakly stratified deep water may be large enough that also the deepest layers may appear partially renewed from the surface. Such lakes do not easily fit into the classification of holomictic or meromictic lakes.

In this publication, we deal with Lake Bosumtwi in Ghana, West Africa, in which deep water is anoxic nearly all year round (Puchniak et al. 2009, Damoah et al. under review). Deep recirculation takes place during the colder and windier rainy season when reduced irradiation (because of clouds) leads to lower surface temperatures and higher wind speeds deliver turbulent kinetic energy to overcome density stratification. Measurements on the circulation during two time windows (2004 to 2006 versus 2018 to 2020) documented changes in the circulation pattern (Damoah et al. under review). Their work indicated that changes in the weather conditions led to reduced deep mixing in recent years (period 2018–2020). While—contrary to what Puchniak stated—traces of oxygen could be detected in the deeper hypolimnion occasionally in rainy seasons of the earlier period (2004–2006), this was not the case in the later period (2018–2020). As a consequence, it could be suspected that Bosumtwi could have accumulated more gases in the deep water than before. Until now, a quantification of dissolved gases has, however, not been attempted so far.

The gases are especially interesting, as fish kills have happened regularly at the time of deeper circulation (Puchniak et al. 2009). It was assumed that dissolved gases in the monimolimnion might be released into the epilimnion and harm the fish. The most probable candidates based on conclusions from past studies were carbon dioxide (CO_2_) and hydrogen sulphide (H_2_S) (Puchniak et al. 2009). Our investigation therefore aimed at (1) identifying and quantifying the various gases present in the deep water, (2) clarifying the accumulation of gases in the deep water of Lake Bosumtwi, and (3) assessing to what extent accumulated gases (or other substances) might have been responsible for fish kills that happened during deep circulation events.

## Methods

### Study site

**Fig. 1:**
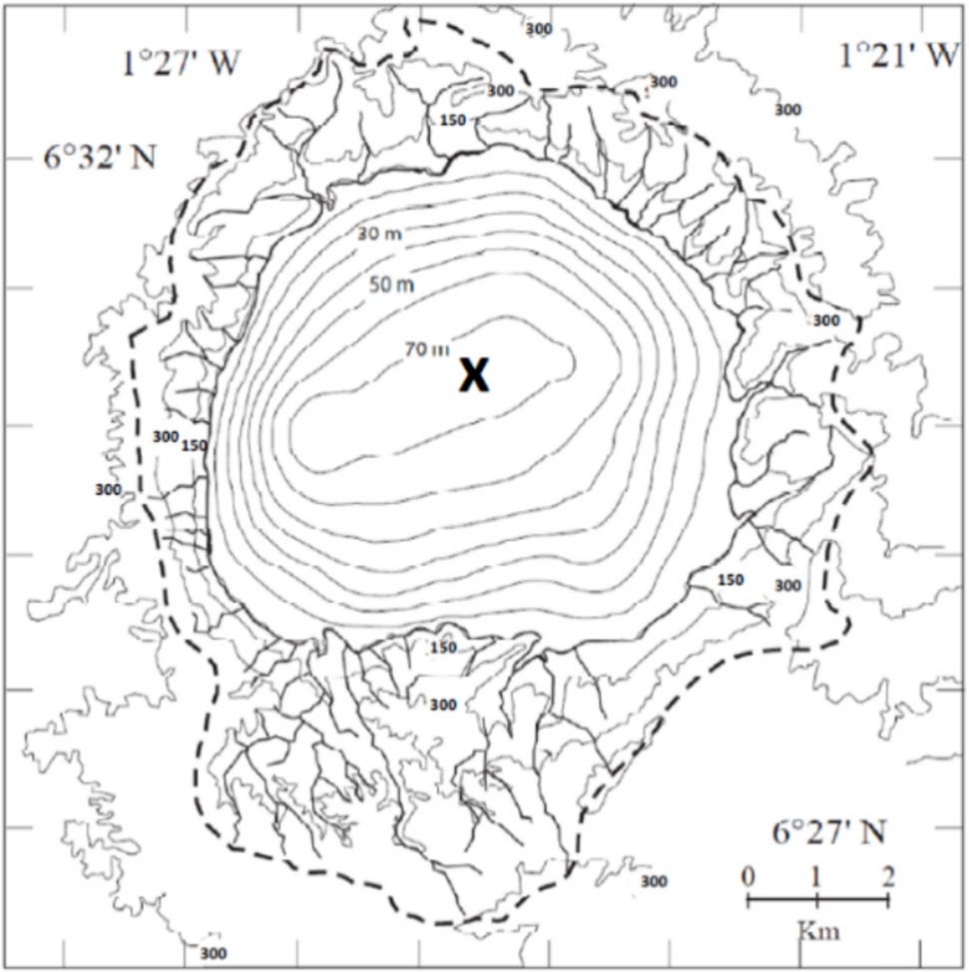
Lake Bosumtwi with depth contours at 10-m interval. Sampling was done at the location of maximum depth N 6.509446 E − 1.410749 marked by “x” (map modified from Otu 2010 and Brooks et al. 2005)

Lake Bosumtwi is a deep, thermally stratified, meso-eutrophic closed tropical lake, occupying a nearly circular depression at an elevation of 99 m above sea level (m.a.s.l.) formed by a meteorite impact about one million years ago (Jones et al. 1981; Koeberl et al. 1997, 2007). It is located in the south-central region of Ghana, West Africa, at 6° 30′ N; 1° 25′ W. High crater walls reaching 460 m.a.s.l. rise from the lake surface. The lake surface area (48.6 km^2^) to catchment area (103.1 km^2^) ratio is large. The latter is characterized by semi-deciduous forest and crop farming. The crater is about 700 m deep, far below the groundwater table, and hence, water flooded the depression. However, groundwater input is highly restricted due to a thick layer of sediment (Koeberl et al. 2007). Sedimentation has filled the lake basin in part and the lake is more than 70 m deep (at the time of producing the map in Fig. 1). Falling water levels may have reduced the maximum depth even further. The lake has regressed and transgressed several times in prehistoric times, and the lowest possible outflow point lies about 110 m above the current lake level (Talbot and Delibrias 1977; 1980). 4000 years ago and before, the local climate was wetter than today and outflows happened occasionally (Talbot and Delibrias 1977, 1980; Talbot et al. 1984). The lake level has dropped since then and continues to do so as a consequence of changing climate conditions.

According to Puchniak et al. (2009), the deepest water in Lake Bosumtwi is permanently anoxic and has a meromictic behaviour: anoxic deep water is present and the deep circulation does not reach the deepest part of the lake (Turner et al. 1996a, b; Puchniak et al. 2009). More recent work by Damoah et al. (under review) showed that traces of oxygen could be detected in the deep water occasionally in the period 2004–2006; however, the most recent measurements in the period 2018–2020 never detected any traces of oxygen in the deep water. The authors concluded that deep mixing was inhibited by changes in the weather over the years between. The thermocline usually was found at a depth of 8 to 10 m during the long-stratified period (between October and June) but fluctuated seasonally to depths of 30–40 m and below, depending on the intensity of convective cooling and wind stress, which drive the seasonal deep circulation and impact biogeochemical properties and their variability within the water column (Puchniak et al. 2009).

Currently, salinity is elevated (about 1.3 mS/cm), as Lake Bosumtwi has been a terminal lake for the last 4000 years. Groundwater seepage into the lake is small, while seepage out of the lake into the groundwater is not expected as the groundwater table lies higher in the surrounding area than the lake level. As a consequence, substances may accumulate in the lake leading to a high load of solutes (including nutrients) in the deep hypolimnion (Puchniak et al. 2009).

Profiles of dissolved inorganic nitrogen (DIN = nitrate + nitrite + ammonium), total phosphorus (TP), and soluble reactive phosphorus (SRP) have been reported to show strong vertical gradients in the water column with high enrichment in the deep, anoxic waters (Turner et al. 1996a; Almond and Hecky 2002; Verburg 2004; Puchniak et al. 2009). In addition, gases such as CO_2_ (average concentration of 59 µmoll^−1^) have been reported in the literature (Puchniak et al. 2009). The usually hypoxic mid-water (depth of 12.5–30 m) inhabits a deep chlorophyll maximum (DCM) that peaks (> 100 µg/L chlorophyll a) during the stratified period (usually in May/June) coinciding with increased lake transparency (Sanful et al. 2017; 2019). The water chemistry is characterized by an average pH of 8.9 (above 9 in surface water above the thermocline), elevated electrical conductivity, and alkalinity of 10,320 µmoll^−1^ (Puchniak et al. 2009).

### Measurements

Measurements were taken during two sampling campaigns in May 2019 and (after a long interval due to Covid travel restrictions) in February 2022, during the rainy and dry seasons respectively and coinciding with strongly stratified lake conditions on both occasions. Deep recirculation typically happens in late August (see above and Damoah et al. under review). Hence, samples from both expeditions were collected when Lake Bosumtwi had been stratified and the last full circulation had happened many years before, at least not since January 2007 (Puchniak, personal observation).

During the campaign in 2019, profiles of temperature, electrical conductivity, oxygen saturation, and pH (also turbidity, fluorescence, and sound speed) were measured with a highly precise multiparameter probe (Sea & Sun Technology, Trappenkamp, Germany). In addition, we used a gas pressure sensor (Pro Oceanus) at a few discrete depths to determine gas pressure as the pressure in a gas space behind a gas-permeable membrane.

After the first campaign had given an initial impression about the general situation, we collected samples for gas analysis in 2022. For the CO_2_ and CH_4_ samples, we equilibrated water samples of 30 ml with 30 ml air and pushed the resulting head space into pre-evacuated exetainers ensuring that the resulting pressure in the exetainers was above atmospheric pressure (for details see Koschorreck et al. 2021). The samples were transported to Germany and measured in the gas chromatograph at the Helmholtz Centre for Environmental Research (UFZ), Department of Lake Research, Magdeburg, Germany. The GC was equipped with a Haysep D column and a flame ionization detector with a methanizer for CO_2_ (SRI-8610C, SRI instruments, Torrance, USA).

In addition, we took water samples for major ions. Major cations were pushed through a 0.45-µm filter on site and measured in the laboratory with an ICP-OES Perkin Elmer 7300 DV according to DIN EN ISO 11885:2009–09. The dissolved anions were measured using an anion chromatographic system (Thermo Fisher ICS 6000) based on DIN EN ISO 10304–1:1995–04. The dissolved organic carbon (DOC) and total organic carbon (TOC) were determined with a DIMATOC®2000 from Dimatec based on the ISO 8245:1999–03 method. Dissolved nitrogen (DNb) and total nitrogen (TNb) were determined photometrically using a Hach Lange system and the norm DIN EN 12260:2003–12. The requested quality of the analytical data was assured by daily routine and the yearly method validation as well as the successful participation on yearly inter-laboratory tests. Based on the ionic composition in the water samples, the density relation could be constructed (Damoah et al. under review). Apart from the main cations, high values of arsenic were detected. As this fact and the assessment and tracing lay beyond the scope of this paper, it was decided to cover this in a separate publication.

We also collected samples for H_2_S, as an unpleasant smell from the lake had been reported during deep recirculation and fish kills. Our samples had a pH of around 8.5, which limited the speed of H_2_S oxidation (see Le et al. 2019). Samples were carried to the Helmholtz Centre for Environmental Research in Germany and then measured in a photometer. The photometer measured total sulphide, of which the toxic H_2_S only forms a portion, which can be quantified from total sulphide and pH measured by the multiparameter probe in situ.

## Results

### Field campaign 2019

**Fig. 2:**
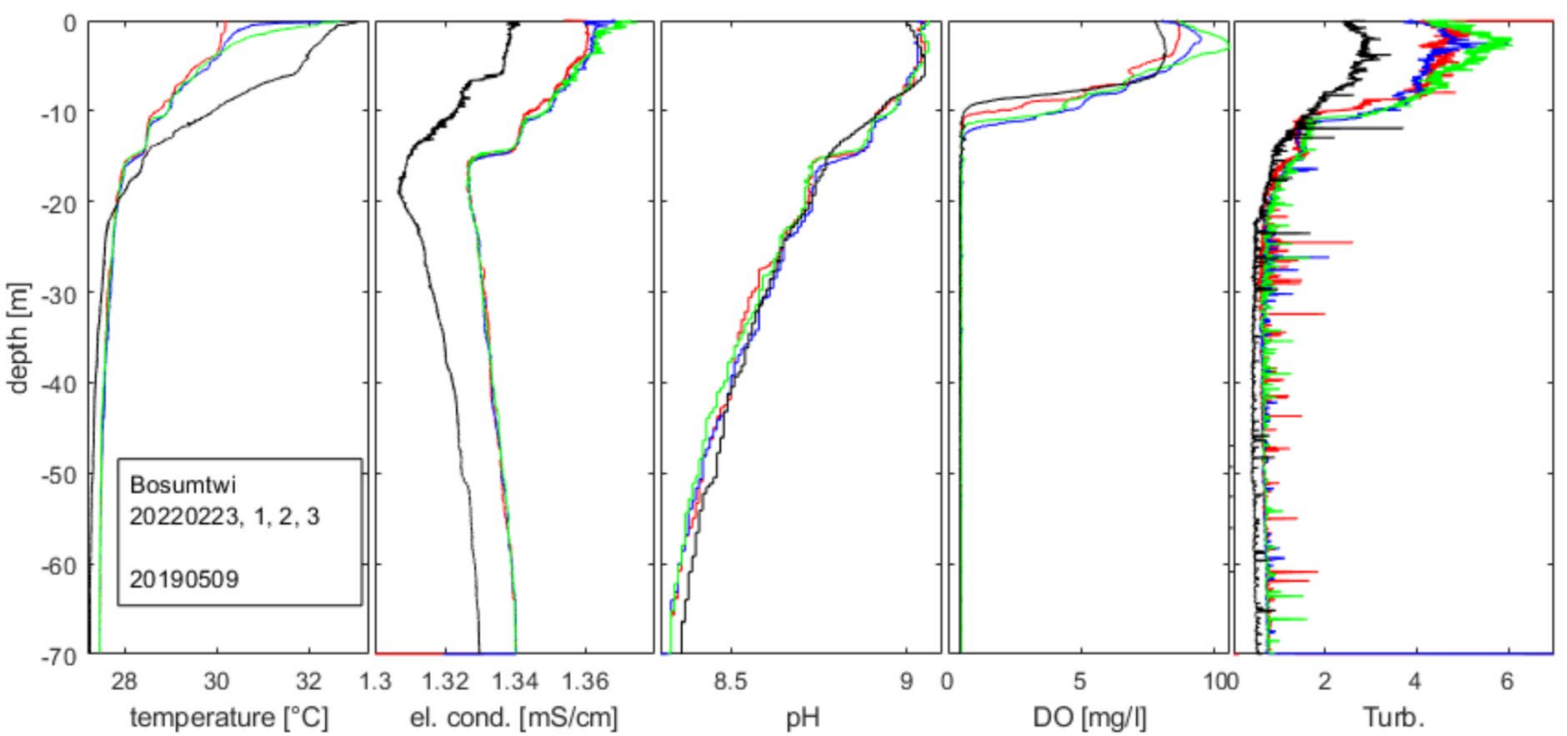
Profiles of Lake Bosumtwi on the dates 9th May 2019 (black) and 23rd Feb. 2022 (colours). From left: temperature, electrical conductivity (compensated to 25 °C), pH, oxygen concentration, turbidity

The field campaign in May 2019 confirmed the stable stratification. Temperatures in the epilimnion reached 32 °C, while the deep water temperature was 27 to 28 °C continuously rising from the lake bed to the surface (Fig. 2). Measurements of electrical conductivity (compensated to 25 °C) showed a minimum at depths around 15 to 20 m and from there rose continuously both towards the surface and the lake bed. The overall variation of electrical conductivity was small (1.31 to 1.34 mS/cm) and hence delivered only a small contribution to the density gradient profile, which was dominated by the temperature gradient.

Values of pH were around 9.1 in the epilimnion falling towards 8.3 at the bottom of the lake. The oxygen concentration in the upper 10 m was close to saturation or even slightly above 100% (~ 108%). Oxygen was not present in the deep water (Fig. 2).

**Fig. 3:**
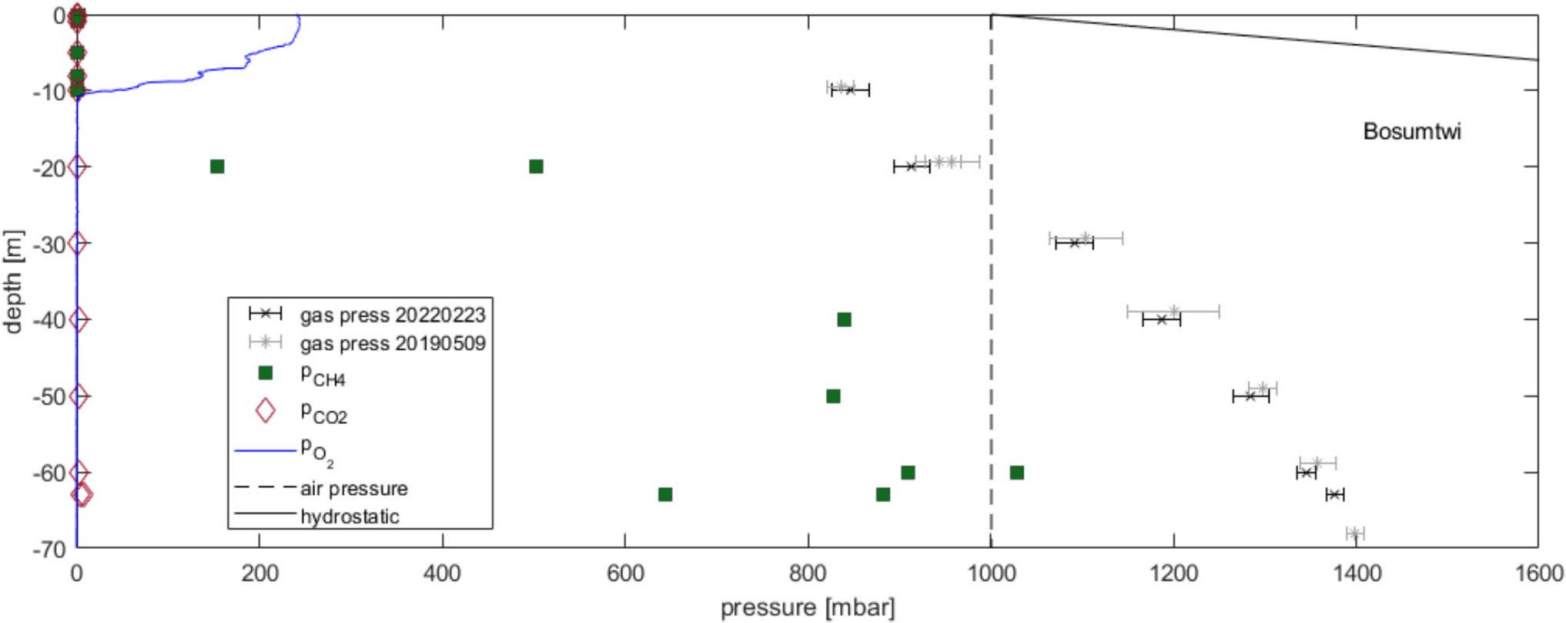
Gas pressures measured in Lake Bosumtwi. Total gas pressure measured in February 2022 (black crosses) and May 2019 (grey stars); partial pressure of methane (green squares) and carbon dioxide (brown diamonds) from samples measured in a gas chromatograph (for more details on measurement and accuracy, see Koschorreck et al. 2021); partial pressure of dissolved oxygen (from multiparameter probe) in relation to average atmospheric pressure at the altitude of Lake Bosumtwi surface (broken line) and hydrostatic pressure (solid line); all samples and measurements (except the 2019 total gas pressure) are from 23rd February 2022

The gas pressure at depths of 10 and 20 m was below atmospheric pressure as expected due to the missing gas pressure of depleted oxygen (compared to atmospheric, Fig. 3). Most of the remaining gas pressure was attributed to dissolved nitrogen (N_2_). In the deep water, gas pressure was above atmospheric and rose to 1400 mbar close to the lake bed. At all depths, the gas pressure was far away from absolute pressure (i.e. hydrostatic plus atmospheric, Fig. 3). Most of the gas pressure above atmospheric nitrogen gas pressure was probably a consequence of methane accumulation.

### Field campaign 2022

In February 2022, our measurements confirmed the situation as in 2019: an epilimnion of 10 m, electrical conductivity minimum at 15 to 20 m depth, oxygen exclusively in the epilimnion, and pH values between 9.1 at the surface and 8.3 at the lake bed. A closer look revealed that the epilimnion in February 2022 was cooler than in May 2019, and conductivity was about 0.012 mS/cm higher over the entire profile (Fig. 2). Over the same time, temperatures rose by about 0.2 °C over the entire deep water body.

The gas pressure measurements agreed with the measurements in 2019 within the expected accuracy (Fig. 3). There was no further accumulation nor any change in the shape of the profile. This was not necessarily expected, as over the 3 years deep recirculation could have happened and released gas from the deep water and accumulation could have resulted in quite differently looking gas pressure profiles. However, this was not observed. In the end, this agreed fully with Damoah et al.’s (under review) observation that the circulation could not reach the deep water in the years between the profiles.

Dissolved methane showed values close to 0 at the surface but rose in the deep water to 800 to 900 mbar (which corresponded to 1.1.. 1.25 mmol/l) in the deep water (Fig. 3). These were clearly elevated values but not spectacular like in Lake Kivu (Boehrer et al. 2019), Lake Monticchio Piccolo (Italy, Fazi et al. 2025), or even Lake Vollert-Süd (Germany, Horn et al. 2017). Carbon dioxide lay significantly higher than atmospheric (partial pressure of about 3 mbar in deep water versus 0.4 mbar in the atmosphere) corresponding to about 0.1 mmol/l in the deep water, but far away from high concentrations as in Lake Nyos (Kusakabe et al. 2008; Boehrer et al. 2021b), Lake Kivu (Boehrer et al. 2019), or Guadiana pit lake (Boehrer et al. 2016).

The chemical composition of major solutes and trace elements in Lake Bosumtwi water was analysed for five depths (listed in Table 1) in both years of observation (2019 and 2022). Measurements of major cations indicated that sodium (Na^+^) was the most abundant representative. At lower concentrations, we found potassium, magnesium, calcium, also ammonium (see below in the paragraph on bound nitrogen), and traces of iron and manganese in the anoxic deep waters. Some chloride was detected throughout the water column while the ion chromatographic determination indicated that sulphate lay below the limit of quantification (0.052 mmol/l). From balancing electrical charges (for 2019 and 2022) and total inorganic carbon (TIC) measurements (only 2019), bicarbonate must have been present in concentrations in the range of 10 mmol/l = 600 mg/l, and hence, it was the most abundant anion. The concentrations from 2019 at 30 m depth were used by Damoah et al. (2025) to find the coefficients of the density formula according to Moreira et al. (2016) and Boehrer et al. (2010):.

1 $${\rho }_{\lambda }\left(T,{k}_{25}\right)={\rho }_{w}\left(T\right)+{k}_{25}\cdot \left[{\lambda }_{0}+{\lambda }_{1}\cdot \left(T-{25}^{o}C\right)\right],$$

where ${\lambda }_{0}=0.584$ and ${\lambda }_{1}=-0.0017$. For the density of pure water ${\rho }_{w}\left(T\right)$, we used the formula supplied by Tanaka et al. (2001).

**Table 1:** Concentrations of solutes in Lake Bosumtwi on 9th or 10th May 2019 and on 23rd Feb. 2022 from water samples analysed in the laboratory. In addition, the following chemical species were below the detection limit at all depths: zinc (< 0.009 mg/l in 2019), aluminium (< 0.02 mg/l in 2019), sulphate (< 5 mg/l 2019 and 2022), sulphide < 0.1 mg/l (2022)

Sample	Depth	Na	K	Mg	Ca	Mn	Fe	Cl^−^	DNb	tNb	DOC	TOC	TIC	El. C
Date	m	mg/l	mg/l	mg/l	mg/l	mg/l	mg/l	mg/l	mg/l	mg/l	mg/l	mg/l	mg/l	mS/cm
2019 05/09 05/10	**0 m**	289	30.5	15.4	8.40	< 0.007	< 0.01	104		0.730			117	1.283
	**15 m**	289	30.4	15.6	8.43	0.009	< 0.01	103		1.360			125	1.254
	**30 m**	275	29.0	15.0	8.21	0.042	0.012	104		2.592			123	1.266
	**50 m**	271	29.2	15.6	8.34	0.055	0.034	103		3.875			127	1.283
	**70 m**	272	29.7	15.4	8.45	0.060	0.049	105		4.411			129	1.286
2022 02/23	**0**	261	26	13.9	7.49	< 0.007	< 0.01	108	0.807	1.32	8.89	9.63		
	**10**	269	27.1	14.5	7.77	< 0.007	< 0.01	107	0.939	1.15	8.67	9.07		
	**30**	264	26.3	13.9	7.72	0.051	0.0267	103	3.38	3.63	8.36	8.34		
	**50**	255	25.7	14.4	7.62	0.05	0.0503	104	4.17	4.48	7.77	8.25		
	**63**	274	27.1	14.5	8.04	0.052	0.049	104	4.51	4.84	8.15	8.49		

Photometer (Hach) measurements of total sulphide sampled and stored under alkaline condition gas free following the EPA Handbook for sampling and Sample Preservation of water and wastewater (EPA-600/4–82-029) lay below the detection limit of 0.1 mg/l = 0.003 mmol/l. Using the pH values from the multiparameter profiles between 8.3 and 8.8 (Fig. 2) compared to the pKs of H_2_S of 7.1 (Worch 2015), we concluded that not more than 6% of total sulphide could be present in the form of H_2_S. This limits H_2_S concentrations to 6% of the detection limit, which amounts to 0.2 μmol/l (= 6 μg/l), clearly below what is considered lethal (Boyd 2014; Cochrane et al. 2019 and citations therein). This corresponded with the sulphate concentration below detection, which would serve as a possible supply of sulphur, which in a chemically reducing environment could be reduced to sulphide.

Nutrient measurements were not attempted due to the long transport of samples to the laboratory in Germany. However, values of bound nitrogen tNb were remarkably high. Although a further analysis of the chemical components of this bound nitrogen was not possible, we were convinced that most of the bound nitrogen was present in the form of inorganic nitrogen. Hence, in the anoxic deep water, we would expect it mainly in the reduced form of ammonium (NH_4_^+^) and ammonia (NH_3_). As the deep water pH values were just below the pKs of NH_4_^+^ (9.25), both species were present; however, the NH_4_^+^ was the clearly dominating form.

Comparing the tNb values of 2022 to 2019, we see a change of 0.5 mg/l in the deep waters (30 m and deeper) over this 3-year interval. Comparing this to the absolute values in the deep water, we can estimate the order of magnitude that is required to accumulate such an amount of tNb to roughly 20 years. As tNb values were probably lower at the time, when deep recirculation still introduced occasionally some oxygen into the deep water (as reported for the period 2004–2006 by Damoah et al. under review), the estimated time agrees well with the perception of accumulating tNb since the last deep mixing events.

## Discussion

**Fig. 4:**
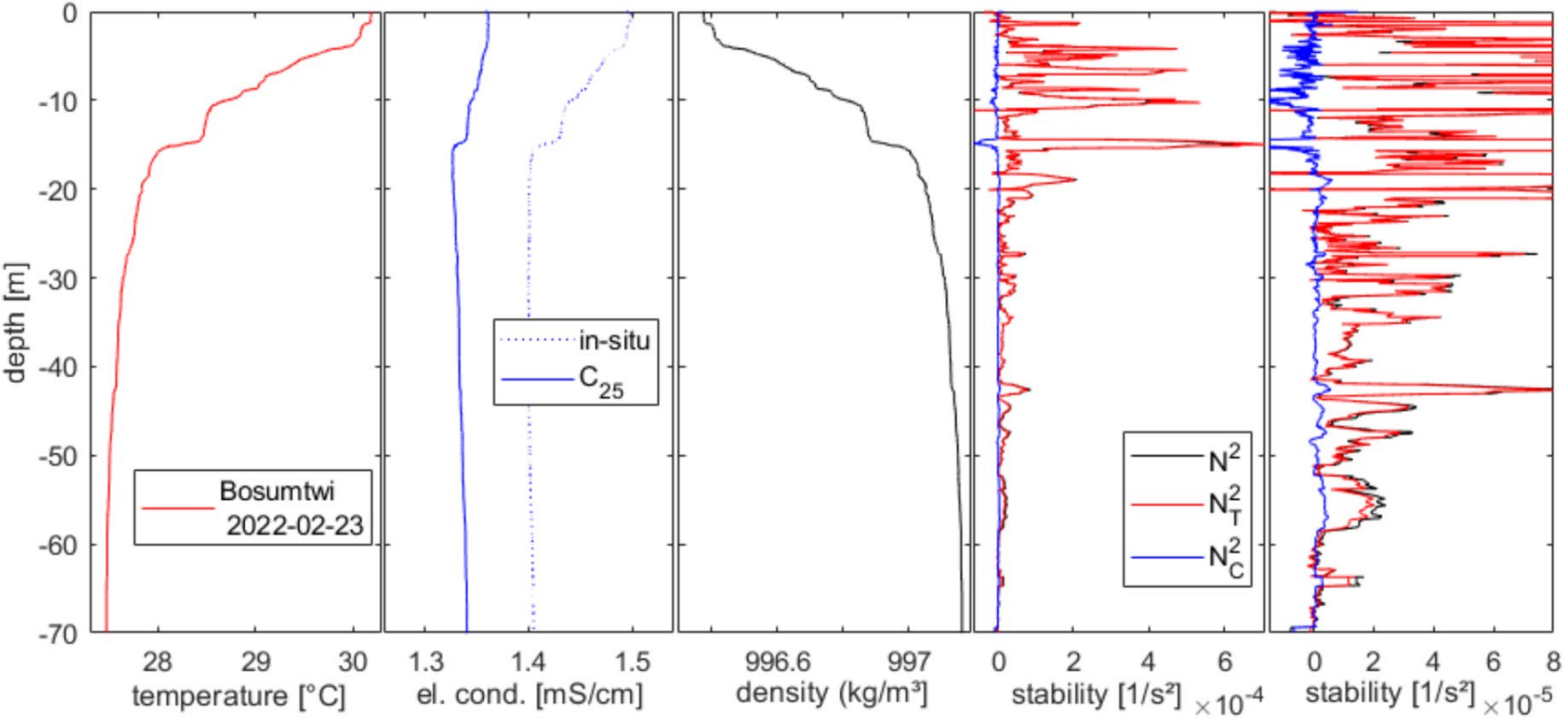
Profiles of quantities connected to density in Lake Bosumtwi from data on 23rd Feb. 2022. From left: temperature, electrical conductivity in situ and compensated to 25 °C, (potential) density following Moreira et al. (2016), stability frequency: total (black) and contribution of temperature gradient (red) and electrical conductivity gradient (blue); same stability profiles at different scale

Our measurements confirmed the stable stratification of Lake Bosumtwi during the hot and dry seasons, as has been reported elsewhere (Damoah et al. under review). We confirmed the oxic epilimnion overlying the anoxic deep water. Density stratification was mainly due to temperature; gradients of solute concentration contributed much less to density gradients. Both temperature and electrical conductivity provided stability; hence, double-diffusive convection (Newman 1976, von Rohden et al 2010) was not expected to happen in Lake Bosumtwi, though we see layers of stratification alternating with layers of no density gradient (Fig. 4, right panel, 30 to 70 m depth). Solutes only contributed marginally to the density gradient in the deep waters.

The increasing electrical conductivity by roughly 0.9% between 2019 and 2022 fitted well with reports of falling water levels by evaporation (compared to rainfall and freshwater inflow), while solutes remained in the lake basin. For a quantitative comparison, the reliable measurements were missing. Doubts about the reliability of the conductivity measurement are easily dispelled by measurements from the campaign by Damoah et al. (under review) of another probe run by UENR (YSI Exo Sonde 2), which documented the same evolution. At the surface, we see the signal of rainy and dry season periods. The mixing reached roughly 30 m in August or September in the years 2018 and 2019. The electrical conductivity however continuously increased from October 2018 without breaking the stratification. Hence, the increasing salinities in the deep water are not directly connected to the falling water level. It seems much more probable that the ions were produced. Table 1 lists that TNb increased by about 0.3 mg/l. If all bound nitrogen appeared as NH_4_^+^, this would make up for a quarter of the additional electrical conductivity. Requiring the same amount of negative charges (anions) indicated that together with other decomposition processes, this conductivity could have been internally produced (calculation following Moreira et al. 2016: density calculator).

The gas pressure profile indicated that both years of investigation were similar in terms of gas content. Methane (and carbon dioxide) had accumulated over time. No change over 3 years indicated that deep mixing did not recirculate the deep water enough to release a large portion of dissolved gases. The gas pressure profile was probably already close to an equilibrium of forming gases and diffusive and occasional small loss by vertical exchange of smaller water parcels. However, as the profile close to the suspected equilibrium was already detected in 2019, this indicated that also the years before 2019 had probably been without full overturn.

The values for methane were remarkable, but not spectacular. Also, carbon dioxide was accumulated, but not to levels of concern. The resulting gas pressure was far away from the absolute pressure profile, and hence, there was no indication of a risk of a large-scale degassing (“limnic eruption”).

**Fig. 5:**
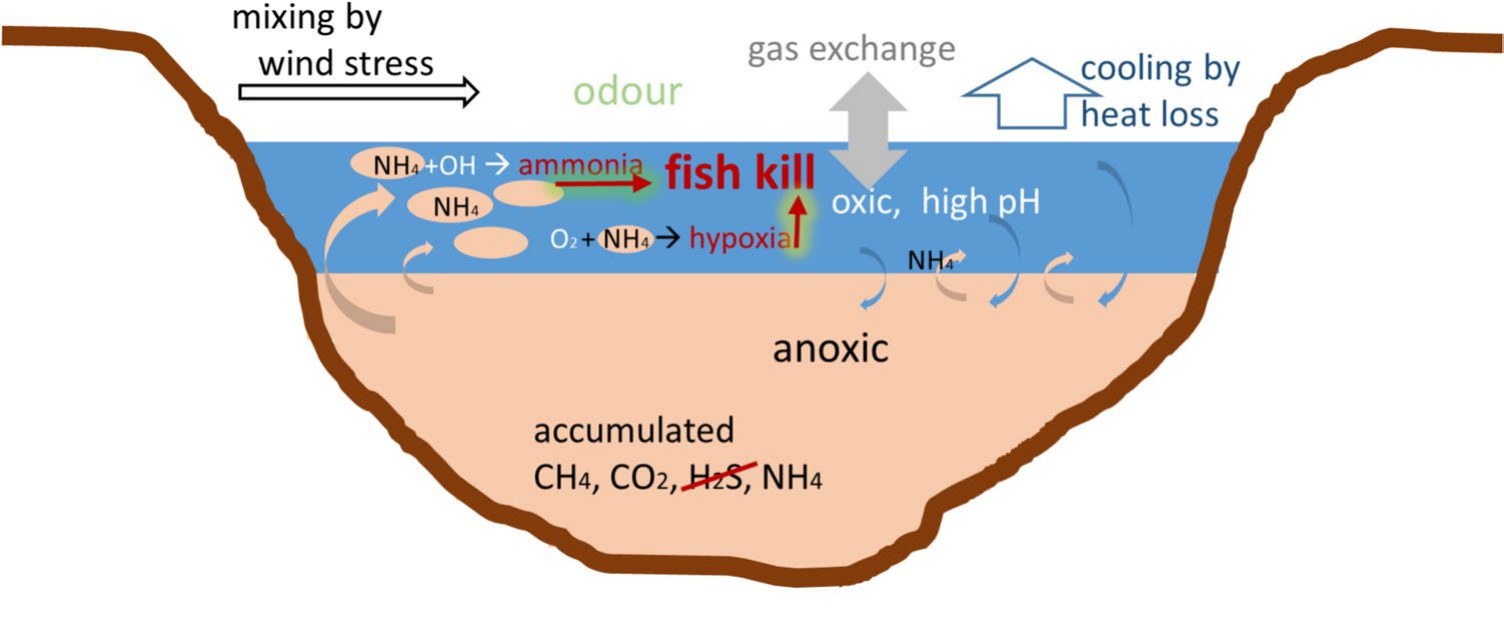
Sketch of Lake Bosumtwi including the processes involved in gas formation and transport and their possible contribution to the fish kills

The fact that, in recent years, fish kills are less intense and less often than reported earlier suited well with observations of Damoah et al. (under review) that deep mixing was significantly reduced compared to the period 2004–2006. With our data, we could not separate this effect from possible overfishing, which also reduced the number of fish living in the lake and hence also the number of potentially killed fish. The question, of which process could have caused fish kills, however, remained. Fish kills had been associated with deep mixing. Hence, we assessed the potentially toxic substances in separate by considering mixing similar volumes of surface water and deep water and checking the resulting water quality for known toxicity for fish. We proceeded from the first suspects to the more complex cases and we included our findings in a sketch of Lake Bosumtwi at the appropriate location (Fig. 5):

1. H_2_S: In the deep water, there was at the most 0.2 µmol/l of H_2_S, as concluded from the detection limit of total sulphide. This is not considered toxic. Mixing water of this H_2_S content into the surface water would even dilute the H_2_S, and finally, the high pH of the surface water would even shift the acid–base-equilibrium towards HS^−^; i.e. even less H_2_S would be in the mixed water. Hence, H_2_S could be excluded from being an important factor for the fish kills in Lake Bosumtwi (see Fig. 5).
2. CO_2_: The concentrations of CO_2_ in the deep water (eight times atmospheric equilibrium) are probably too low to be responsible for fish kills. Mixing into the surface water would result in even lower concentrations. In conclusion, CO_2_ was not a major player in the fish kills.
3. O_2_ dilution: Equal parts of deep water mixed into the surface water would reduce the oxygen content to half; this would definitely be no good situation for fish; however, a large-scale fish kill (through this process alone) would require a larger amount of deep water compared to surface water and the mixing needed to be completed in a time frame faster than the replenishment of oxygen from the atmosphere. Oxygen dilution could endanger the fish in Lake Bosumtwi, but it needed to happen fast and include nearly all deep water within a short period.
4. Ammonia from bound nitrogen: We found 4.84 mg/l = 0.34 mmol/l total nitrogen bound (TNb) in the deepest sample and 4.48 mg/l = 0.32 mmol/l in the 50-m sample. At pH 8.3 to 8.5 as in the deep water (Fig. 2), nearly all TNb was expected in the form of NH_4_^+^ and only a little NH_3_. Mixing deep water into surface water at equal ratios would dilute the TNb by a factor of 2, but would bring the pH close to the pKs of NH_4_^+^. This would result in about half the TNb in the form of NH_3_: i.e. concentrations of 0.08 mmol/l could be produced. This concentration would probably be toxic for many fish species (Ip et al. 2001; Randall and Tsui 2002; Thurston and Russo 2002; Levit 2010).
5. Oxygen depletion through total nitrogen bound: The surface water contained about 8 mg/l O_2_ = 0.25 mmol/l O_2_. Mixing at equal parts, deep water would dilute oxygen to 0.125 mmol/l; the total nitrogen bound (TNb) would be diluted to 0.17 mmol/l. Oxidizing NH_3_ or NH_4_^+^ would require between 1 and 2 mol O_2_ per mol TNb, depending on which end products were assumed. This effect has the potential to reduce the oxygen in the water enough to kill fish.

It is clear that our assessment can only be based on the recent period from which we have proper data about circulation and chemistry. As the deep recirculation has ceased, the accumulation of substances in the deep water has probably increased (e.g. Boehrer et al. 2017; Gulati et al. 2017). Hence, the arguments against H_2_S and CO_2_ as crucial factors remain valid.

The relevance of the TNb for the ecology of Lake Bosumtwi raises the question of its origin. We expect a contribution from decomposed organic material. From Damoah et al. (under review), we know that deep recirculation has not reached the deepest parts of the lake and the period without deep mixing extends back several more years. In conclusion, the TNb could have accumulated over a few years (e.g. Boehrer et al. 2017; Gulati et al. 2017). This results in production rates that lie within reported high values measured in experiments with sediments from the Baltic Sea (Liesirova et al. 2023).

From our measurements, we know that total gas pressure amounts to 1200–1400 mbar below 40 m depth (Fig. 3). Out of this total gas pressure, 800–900 mbar must be attributed to methane (Fig. 3) leaving a gas pressure of 400–500 mbar for N_2_. In equilibrium with the atmosphere, we would expect 780 mbar of N_2_ gas pressure. A rough estimate from the gas pressures above indicated that the deep water may be short of around 250 mbar N_2_ gas pressure (one-third) compared to the atmospheric equilibrium. This is tantamount to 0.32 mmol/l of N or 4.5 mg/l of TNb. Where has the N_2_ gone? A temperature effect in the formation of deep water as in Lake Kivu (Schwenk et al. 2022a, 2022b) is very improbable. Unfortunately, no direct measurements of N_2_ are available to confirm this fact. This is in part a consequence of difficulties to avoid air contamination on the long transport into the laboratory and other technical issues (see e.g. Bärenbold et al. 2020). As a consequence, only a few accurate measurements of N_2_ in monimolimnetic waters exist (e.g. Vollert-Sued: Horn et al. 2017, Laghi Monticchio: Fazi et al. 2025). There are good reasons to close this knowledge gap in Lake Bosumtwi and other lakes.

## Conclusions

Lake Bosumtwi is a tropical lake, which does not experience extended periods of full overturn. Measurements confirm an anoxic deep water body. Gases accumulate there and gas pressure is elevated. We find a reducing chemical environment. Accumulated gases in the deep water, reduced nitrogen gas pressure, and very high values of total nitrogen bound (TNb) concur with observations (Damoah et al. under review) that deep recirculation has not happened over the last few years. Though gases (mainly methane) have accumulated in Lake Bosumtwi’s deep water, gas pressure does not pose a danger in terms of limnic eruptions.

The investigation aimed at quantifying dissolved gases in the deep water and determining their possible involvement in previous fish kills. We did not detect any hydrogen sulphide (H_2_S), nor can carbon dioxide (CO_2_) be responsible for fish kills at the measured concentrations (of 3 mbar in the deep water). The dilution of oxygen (O_2_) in the surface water by anoxic deep water during periods of deep mixing will be a danger for the fish, but it is questionable whether the dilution alone can be responsible for fish kills. The potential for fish kills is most probably connected to the extremely high concentrations of total nitrogen bound TNb. This works on two paths: firstly, ammonium (as a large part of TNb) is transferred into toxic ammonia when mixed into high pH surface water; secondly, the TNb from the deep water can require most of the dissolved oxygen in the surface water, which—together with the dilution effect—can cause distress to fish and eventually kill them.

## Data Availability

Original data are available on request from Bertram.Boehrer@ufz.de.
